# Intramyocardial Adipose-Derived Stem Cell Transplantation Increases Pericardial Fat with Recovery of Myocardial Function after Acute Myocardial Infarction

**DOI:** 10.1371/journal.pone.0158067

**Published:** 2016-06-23

**Authors:** Jong-Ho Kim, Soon Jun Hong, Chi-Yeon Park, Jae Hyung Park, Seung-Cheol Choi, Sang-Keun Woo, Jung Woo Yu, Gi Jeong Cheon, Hyung Joon Joo, Do-Sun Lim

**Affiliations:** 1 Department of Cardiology, Cardiovascular Center, College of Medicine, Korea University, Seoul, Republic of Korea; 2 Molecular Imaging Research Center, Korea Institute of Radiological and Medical Sciences (KIRAMS), Seoul, Republic of Korea; 3 Department of Nuclear Medicine, Cancer Research Institute, Seoul National University Hospital, Seoul, Republic of Korea; Indiana University School of Medicine, UNITED STATES

## Abstract

Intramyocardial injection of adipose-derived stem cells (ASC) with other cell types in acute myocardial infarction (AMI) animal models has consistently shown promising clinical regenerative capacities. We investigated the effects of intramyocardial injections of mouse ASC (mASC) with mouse endothelial cells (mEC) on left ventricular function and generation of pericardial fat in AMI rats. AMI rat models were created by ligating left anterior descending coronary artery and were randomly assigned into four groups: control (*n* = 10), mASC (*n* = 10), mEC (*n* = 10) and mASC+mEC (*n* = 10) via direct intramyocardial injections, and each rat received 1x10^6^ cells around three peri-infarct areas. Echocardiography and cardiac positron emission tomography (PET) were compared at baseline and on 28 days after AMI. Changes in left ventricular ejection fraction measured by PET, increased significantly in mASC and mASC+mEC groups compared to mEC and control groups. Furthermore, significant decreases in fibrosis were confirmed after sacrifice on 28 days in mASC and mASC+mEC groups. Successful cell engraftment was confirmed by positive Y-Chromosome staining in the transplantation region. Pericardial fat increased significantly in mASC and mASC+mEC groups compared to control group, and pericardial fat was shown to originate from the AMI rat. mASC group expressed higher adiponectin and lower leptin levels in plasma than control group. In addition, pericardial fat from AMI rats demonstrated increased phospho-AMPK levels and reduced phospho-ACC levels. Intramyocardial mASC transplantation after AMI in rats increased pericardial fat, which might play a protective role in the recovery of myocardial function after ischemic myocardial damage.

## Introduction

Ischemic cardiac diseases such as acute myocardial infarction (AMI) are a leading cause of death worldwide. Despite recent advances in clinical treatment for such diseases, the mortality rate associated with a failing heart remains very high. Inducing recovery of damaged myocardium has become an important therapeutic goal for treating AMI patients. Therefore, cardiac repair including regeneration of damaged left ventricular (LV) function through stem cell-based transplantation deserves much attention. Stem cell transplantation therapy has been used to treat failing hearts for more than a decade, and a large number of studies have provided profound evidence that implanted cells can participate in generation of new tissue to mechanically and biochemically support the damaged heart and thus enhance its function [1, 2].

Several studies using various types of stem cells have shown promising results in halting myocardial damage and improving LV function [3, 4]. Among many different stem cells, regenerative abilities of adipose-derived stem cells (ASC) are thought to be a practical cell source for cell-based transplantation due to ease of isolation from patients, less invasive collection, large quantities of autologous cells, multi-potency to differentiate into multi-lineages, developmental plasticity, and avoidance of ethical issues [5]. A number of preclinical studies have demonstrated treatment of damaged myocardium with ASC in combination with other cell types in order to maximize efficacy, and implanting ASC with endothelial cells (EC) provides efficient blood supply through formation of vasculature [6].

Increased pericardial fat is frequently observed in coronary artery disease (CAD) or in obese patients and has been implicated as an important risk factor of coronary atherosclerosis [7, 8]. Excessive increase in pericardial fat layer is known to interfere with punctual contraction and relaxation of myocardium and is associated with deposition of excess cholesterol within underlying arteries. However, pericardial fat is also considered to exert local effects as a paracrine organ by secreting various adipokines, and low adiponectin levels in plasma has been associated with progression of CAD and higher incidence of AMI [9, 10]. In this study, we investigated the effects of intramyocardial injections of ASC with EC and their effects on LV function after AMI in rats, in addition to the role of pericardial fat generated after ASC intramyocardial injections.

## Materials and Methods

### Animal care

All procedures were approved by the Korea University Institutional Ethics Committee for Animal Research (KUIACUC-2014-110). All animals were maintained in a specific pathogen-free (SPF) grade animal room at constant temperature of 22°C ± 1°C and 50% ± 5% humidity with 12-hour light/dark cycles. All researches complied with the guidelines from the US National Institutes of Health and Directive 2010/63/EU of the European Parliament on the protection of animals used for scientific purposes.

### Cell sources

Mouse adipose-derived stem cells (mASC) were isolated from male ICR albino mice (Orient Experimental Animal Laboratory, Gyeonggi, Republic of Korea). The mice were given an intraperitoneal (IP) injection anesthesia with a mixture of ketamine (44 mg/kg; Yuhan) and xylazine hydrochloride (0.75 mg/kg; Rompun, Bayer), and euthanized. Mouse inguinal fat pads were dissected, minced, and digested with 0.1% Type I collagenase (#4197, Worthington Biochemical) for 45 mins at 37°C. The equal volume of culture medium containing 10% fetal bovine serum (FBS; #16000–044, Invitrogen) was added, followed by filtration through 100 μm nylon mesh (352360, BD). Then the samples were washed with PBS via centrifugation at 600 g, and the cells were suspended and incubated in 160 mM NH_4_Cl for 10 mins. The cells were plated in a 100-mm culture dish and maintained in Mesencult basal medium, supplemented with MSC stimulatory supplements (#05501, StemCell Technologies) and 100 unit/mL penicillin and 100 μg/mL streptomycin (P/S; #15140, Invitrogen). The cells were incubated at 37°C in a humidified incubator with 5% CO_2_. Culture media were changed daily and mASC were used at passage 3 to 5 for *in vitro* experiments and at passage 8 to 10 for *in vivo* experiments.

Mouse endothelial cells (mEC) isolated from the lungs of mice were purchased from Cell Biologics (#CD-1011L). Initially, 1 × 10^6^ cells were plated in a 100-mm culture dish and cultured with Dulbecco’s modified Eagle’s medium-low glucose medium (DMEM-LG; #11885–092, Invitrogen), containing 10% FBS and P/S. Medium was changed every 2 days and mEC were used at passage 3 to 5 for *in vitro* experiments and at passage 9 to 11 for *in vivo* experiments.

### Immunofluorescence staining

Determination of surface antigens for CD31 (553370, BD), CD34 (16-0341-85, eBioscience), CD106 (553330, BD), and Sca-1 (557403, BD) were achieved by fluorescence staining and counterstaining for 4',6-diamidino-2-phenylindole (DAPI; D9542, Sigma). Sequential steps of cell fixation in 4% paraformaldehyde (PFA; P6148, Sigma), blocking with 5% normal goat serum (NGS; #16210, Invitrogen), then washing with 1X PBS + 0.1% Tween 20 (PBST; P2287, Sigma) were performed, followed by 1 hr incubation with primary antibodies for candidates described earlier. After PBST washing, secondary antibodies incubation using Alexa Fluor-594 conjugated anti-rat (A11007, Molecular Probes) antibodies were carried out for 1 hr. Nuclei were counterstained with DAPI, and the cells were mounted with fluorescence mounting medium (S3023, DAKO). Fluorescence images were obtained using a TE-FM Epi-fluorescence system attached to an Olympus BX61 inverted microscope.

### Flow cytometric analysis

Cell suspensions were fixed in 4% PFA then fluorescence-stained for CD31, CD34, CD106, and Sca-1 for 20 mins at room temperature (RT) for additional confirmation of cellular characteristics. Following incubation of primary antibodies, secondary antibodies containing PE-labeled anti-rat IgGs (#12-4822-87, eBioscience) were used.

To elucidate the origin of pericardial fat, briefly, the isolated pericardial fats from rat hearts were minced and digested as mentioned above. After adding the equal volume of culture media containing FBS, the samples were filtered with 100 μm nylon meshes and washed with PBS. The red blood cells were lysed with the NH_4_Cl solution, then samples were stained with mouse-specific primary antibodies against CD34, CD44, CD106, CD140a (135902, Biolegend), and Sca-1. Also, rat-specific primary antibodies against CD31 (550300), CD44 (554869), CD54 (554967), CD106 (559165, all from BD), and CD140b (ab32590, Abcam) were used. Following each primary antibody incubation, secondary PE-labeled anti-rat IgGs, FITC-labeled anti-mouse (#11-4011-85), and anti–rabbit IgGs (#11-4839-81, both from eBioscience) were applied. Flow cytometry analysis was performed using a flow cytometer and Cell Quest Pro software (FACS Calibur, BD).

### Differentiation potential of mASC

Twenty thousand of mASC were plated into 12-well plates containing 0.1% gelatin (G9391, Sigma)-coated glass coverslips and cultured in culture medium for 3 days. Adipogenic differentiation of mASC was induced by incubation in culture medium with 1 μM dexamethasone (D1756), 100 μM indomethacin (I7378), 0.5 μM methyl-isobutylxanthin (I7018), and 10 μg/mL insulin (I2643) for 10 days. After 10 days of culture, Oil Red O (O1391, all from Sigma) staining was employed to assess the degree of lipid accumulation within the cells. Osteogenic differentiation of mASC was induced by incubation in culture medium with 1 μM dexamethasone, 10 mM glycerophosphate (G6501), and 50 μM ascorbic acid (A5960) for 21 days. Eventually, osteogenic differentiation was determined by Alizarin Red S (A5533, all from Sigma) staining. Endothelial differentiation of mASC was induced by incubation in 60% culture medium with 40% MCDB-201 (M6770), 10^−8^ M dexamethasone, 10^−4^ M ascorbic acid, linoleic acid (L1012, all from Sigma), insulin-transferrin-selenium-pyruvate supplement (ITSP; LS030-01, Welgene), P/S, and 20 ng/mL vascular endothelial growth factor (VEGF; 493-MV, R&D Systems) for 21 days. For cardiomyogenic differentiation, 2 × 10^4^ of mASC were plated into 6-well plates containing 0.1% gelatin-coated glass coverslips and cultured in modified cardiomyogenic differentiation medium [MCM: DMEM-LG, 10% fetal calf serum (FCS), 1% P/S, insulin-transferrin-selenium supplements (41400–045, Gibco), 50 mg/mL BSA, 0.47 μg/mL linoleic acid, 10^−4^ Mascorbicacid,10^−9^ Mdexamethasone] for 3 days. Cardiomyogenic differentiation of mASC was induced by incubation in MCM for 21 days. At day 14 and 21 of cardiomyogenic differentiation, mASC were treated with 100 ng/mL trichostatin A (TSA; T8552, Sigma) for 24 hrs [11].

Endothelial and cardiomyogenic differentiation was assessed. mASC were incubated overnight at 4°C with primary antibodies; anti-Flk-1 (sc-6251), anti-α-actinin (sc-15335, both from Santa cruz), and anti-cardiac troponin T (cTnT; RV-C2, DSHB). Then, the cells were stained with Alexa Fluor-594 conjugated anti-rat and anti-mouse or Alexa Fluor-488 conjugated anti-mouse antibodies for 1 hr at RT. DAPI staining was applied to counterstain nuclei. Fluorescence images were obtained using a TE-FM Epi-fluorescence system attached to an Olympus BX61 inverted microscope.

### Quantitative RT-PCR

Total RNAs were extracted from mASC using Trizol reagent (TR118, MRC). cDNA synthesis and RT-PCR were performed. The primers used for quantitative RT-PCR were as follows: 5'-AGAGACGGTCTTGTCGCAGT-3', 3'-TACTGGGCTTCGAGAGCATT-5' (CD31); 5'-CTCCCCTCCTGAGGCAAT-3', 3'-CGTGGAGGAGCTGATCTTG-5' (CD144); 5'- GGCTCACTTCGAGAACAGGA-3', 3'-TCATTGCGAATACGCTGCT-5' (cTnT); and 5'-GGTGAAGAAGGAGGACATTGA-3', 3'-ATGGCTCAGCCCTCAAACT-5' (cTnI). Quantitative RT-PCR data were pooled from three independent experiments. Relative gene expression levels were quantified based on Ct and normalized to the reference gene, GAPDH (5'-TTCACCACCATGGAGAAGGC-3', 3'-GGCATGGACTGTGGTCATGA-5').

### AMI model and cell transplantation

Female Sprague-Dawley (SD) rats weighing 180 to 200 g were purchased from Orient Experimental Animal Laboratory (Gyeonggi, Republic of Korea). The SD rats were given an intraperitoneal (IP) injection anesthesia with a mixture of ketamine (60 mg/kg) and xylazine hydrochloride (7.5 mg/kg). An 18-gauge angiocatheter was utilized as an intubation tube throughout the procedure. The left coronary artery of the heart was ligated with a 6–0 silk suture, 5 mm from the left coronary atrial appendage. After confirmation of the presence of AMI, 1 × 10^6^ of mASC, 1 × 10^6^ of mEC, or 5 × 10^5^ mASC + 5 × 10^5^ mEC suspension in 100 μL of culture medium was injected at three peri-infarct areas. Culture medium in 100 μL was injected as a control treatment. After cell transplantation, the chest wall, muscle layers, and skin were closed with 3–0 silk sutures. All rats were continuously monitored after surgery. All rats were provided an IP injection of an analgesic agent, ketoprofen (5 mg/kg) after surgery and monitored at least twice a day for the first 7 days and at least once a day until 28 days after surgery. For sacrifice, rats were euthanized by IP injection with the mixture of ketamine (60 mg/kg) and xylazine hydrochloride (7.5 mg/kg).

### Echocardiographic analysis

Echocardiography was performed with a commercially available echocardiography system (Vivid 7, GE Medical Systems) with a 10 MHz small linear array transducer (i13L, GE Medical Systems) [12]. Animals were anesthetized with a mixture of ketamine (60 mg/kg) via intramuscular (IM) injection. All measurements were taken over two consecutive cardiac cycles and averaged. All measurements were performed by an experienced cardiologist who was blinded to the study groups. Each value was the average of two measurements. The percentage of left ventricular fractional shortening (LFFS) was calculated using M-mode. Left ventricular ejection fraction (LVEF) was calculated as: LVEF (%) = (diastolic volume–systolic volume) / diastolic volume. End diastolic volumes and end-systolic volumes were calculated by manually drawing endocardial contours at end-diastolic and end-systolic phases in the apical two chambers view using the modified Simpson’s rule. Moreover, wall motion score index (WMSI) was measured by echocardiography to assess functional improvement in segmental ventricular wall motion. Echocardiographic evaluations were repeated on 1 and 28 days.

### Small animal positron emission tomography imaging

Cardiac positron emission tomography (PET) study was performed under anesthesia by 1–4% isoflurane inhalation (Forane, Choogwae) in oxygen with a small animal PET scanner (Inveon^TM^, Siemens Preclinical Solutions) during 20 mins after 1 hr uptake of ^18^F-FDG. PET images were reconstructed using an ordered subset expectation maximization 2D algorithm. Rat PET cardiac images were reoriented to short-axis images and converted into DICOM format. Polar maps were generated by the QGS software (Cedars QGS 2008, Siemens) [13]. Myocardial infarct size was defined by the percent infarction area of the total LV myocardium. LVEF rate was calculated from gated PET images.

### Histological examination after transplantation

At 28 days, rats were sacrificed and their hearts were fixed with 4% PFA and then embedded in paraffin wax for sectioning. Paraffin-embedded hearts were sectioned from the infarcted LV wall and the septum at 5 or 10 um thickness.

Masson's Trichrome (MT) staining was performed using the Trichrome stain kit (HT15) with following modifications: nuclei were stained with Celestine Blue solution followed by Gill’s hematoxylin (GHS316) staining, and tissue was incubated for 1 hr in Bouin’s solution (HT10132) before muscle staining with Biebrich scarlet-acid Fuchsin (HT151, Sigma). For fluorescence *in situ* hybridization (FISH), prepared tissue slides were dehydrated and incubated with a probe specific to whole mouse Y-Chromosome (Y-Chr; IDMR1056, Labs Biotechnology) for 10 hrs at 37°C in a humidified chamber. Tissues were then incubated with DAPI. Quantification was achieved using Image-Pro 7.0 software (Mediacybernetics).

Fat staining was studied with lipid fixation in a paraffin-embedded section [14]. Collected samples were blocked in 5% normal goat serum (NGS; #16210, Gibco), followed by fluorescence staining for FAT/CD36 (MA1-40243, Thermo scientific) for 2 hrs at room temperature (RT). Following incubation with primary antibodies, samples were stained with secondary Alexa Flour 594-conjugated anti-mouse (A11005, Molecular probes) antibodies. Nuclei were counterstained with DAPI.

Angiogenesis was assessed by staining thin sections with antibodies for smooth muscle α-actin (α-SMA; A2547, Sigma). After immunostaining with antibodies for α-SMA, the sections were incubated with biotinylated anti-mouse IgG for 30 mins at RT, followed by ABC treatment (PK-6100). After color development with 3,3'-diaminobenzidine (DAB) substrate (SK-4100, both from Vector). The number of vessels on 30 sections (two fields per section, three sections per heart, *n* = 5 for each group) were counted α-SMA-positive vessels in the posterior wall, border, and infarct zone.

All digital images were obtained using a TE-FM Epi-fluorescence system attached to an Olympus BX61 inverted microscope. Images of sections were photographed at X400 magnification and analyzed by a trained blinded reader.

### Enzyme-linked immunosorbent assay (ELISA)

Peripheral blood samples were collected from tail veins on a weekly basis using a 24-gauge catheter (382412, BD). The Blood plasma was separated using Ficoll (#17-1440-03, GE Healthcare). Adiponectin and leptin levels were evaluated with Rat total Adiponectin/Acrp30 (RRP300, R&D Systems) and Leptin (ELR-Leptin-001, Ray biotech) enzyme-linked immunosorbent assay (ELISA) kits.

To evaluate FGF-21 levels secreted from mASC and mEC, 1. 5 X 10^5^ of mASC and 1. 5 X 10^5^ of mEC were plated on 6-well plates. After 24 hrs of culture, FGF-21 supernatant levels were determined by Mouse FGF-21 (MF2100, R&D Systems) Immunoassay kits. The ELISA reaction product was quantified by measuring absorbance at 450 nm and 540 nm using an ELISA reader (Spectra Max M2e), and data were analyzed using SoftMax Pro (both from Molecular Devices).

### Western blotting

Pericardial fat tissue in lysis buffer (#9803S, Cell signaling) containing 1 mM PMSF was homogenized (Precellys 24, Bertin Technologies) twice for 20 sec at 6,000 rpm, with a 20 sec pause between the homogenization steps. Cell lysates were subjected to sodium dodecyl sulfate polyacrylamide gel electrophoresis (SDS-PAGE) and transferred onto a polyvinylidene difluoride (PVDF) transfer membrane. The membranes were stained using following primary antibodies: anti-5'-adenosine monophosphate-activated protein kinase α (AMPKα; #2603), anti-phospho-AMPKα (pAMPKα; #2535), anti-Acetyl-CoA carboxylase (ACC; #3676), anti-phospho-ACC (pACC; #3661, all from Cell Signaling), and anti-glyceraldehyde 3-phosphate dehydrogenase (GAPDH; G8795, Sigma). These membranes were incubated with horseradish peroxidase-linked anti-rabbit and horseradish peroxidase-linked anti-mouse anribodies (SC-2005, Santacruz), followed by detection with enhanced chemiluminescence Western blotting substrate (#32106, Thermo scientific) exposed to X-ray film.

### Quantification and statistical analysis

All statistical values were expressed as mean ± standard deviation (SD). Association was analyzed by Pearson correlation analysis for normal distribution or Spearman correlation analysis for non-normal distribution. Analysis of variance (ANOVA) was used to compare normally distributed data from all groups, and *p < 0.05 was considered to be statistically significant. Significant differences between means were determined by ANOVA followed by Student-Newman-Keuls test. All statistical analyses were performed using Sigma Stat 3.1 (Systat) and SAS 9.4 (SAS institute) software.

## Results

### Characterization of mASC and mEC

**Fig 1:**
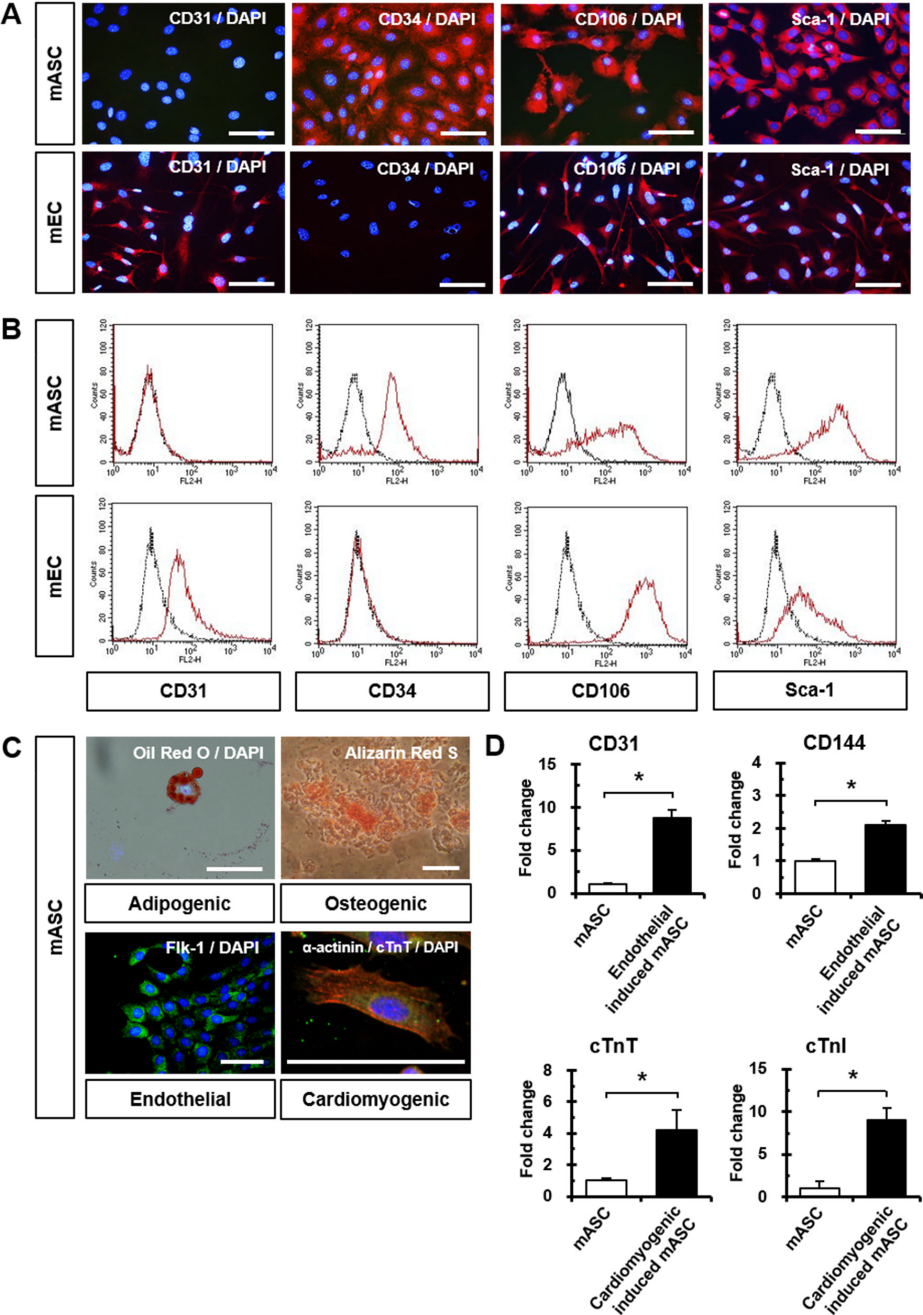
Characterization of mASC and mEC. (A) Comparison of immunofluorescence images showing CD31, CD34, CD106, and Sca-1 expression in mASC and mEC. Nuclei were stained with DAPI. (B) Flow cytometry analyses of CD31, CD34, CD106, and Sca-1 in mASC and mEC. (C) Adipogenic, osteogenic, endothelial, and cardiomyogenic differentiation in mASC. Nuclei were stained with DAPI. (D) Quantitative RT-PCR results of endothelial and cardiomyogenic differentiation with endothelial (CD31 and CD144) and cardiomyocyte-specific markers (cTnT and cTnI). *p < 0.05.

The cellular characteristics of mASC and mEC were studied by fluorescence immunostaining, and positive surface antigen expressions of CD31, CD34, CD106, and Sca-1 were confirmed. mASC were positive for CD34, CD106, and Sca-1, and mEC were positive for CD31, CD106, and Sca-1 (Fig 1A). In addition, CD54, CD102, CD105, and CD144 surface antigens were expressed on mEC (data not shown). Flow cytometry results were in agreement with fluorescence immunostaining results (Fig 1B). The differential potential of mASC was investigated by inducing mASC into four different cell types. Adipogenic, osteogenic, endothelial, and cardiomyogenic differentiation of mASC were confirmed by Oil Red O, Alizarin Red S staining, Flk-1, α-actinin and cardiac troponin T (cTnT) expression, respectively (Fig 1C). mASC exhibited significantly higher endothelial (CD31 and CD144; 8.71-fold and 2.10-fold) and cardiomyogenic (cTnT and cTnI; 4.18-fold and 9.06-fold) differentiation potentials compared to undifferentiated mASC (Fig 1D).

### Evaluation of cardiac function after cell transplantation

**Fig 2:**
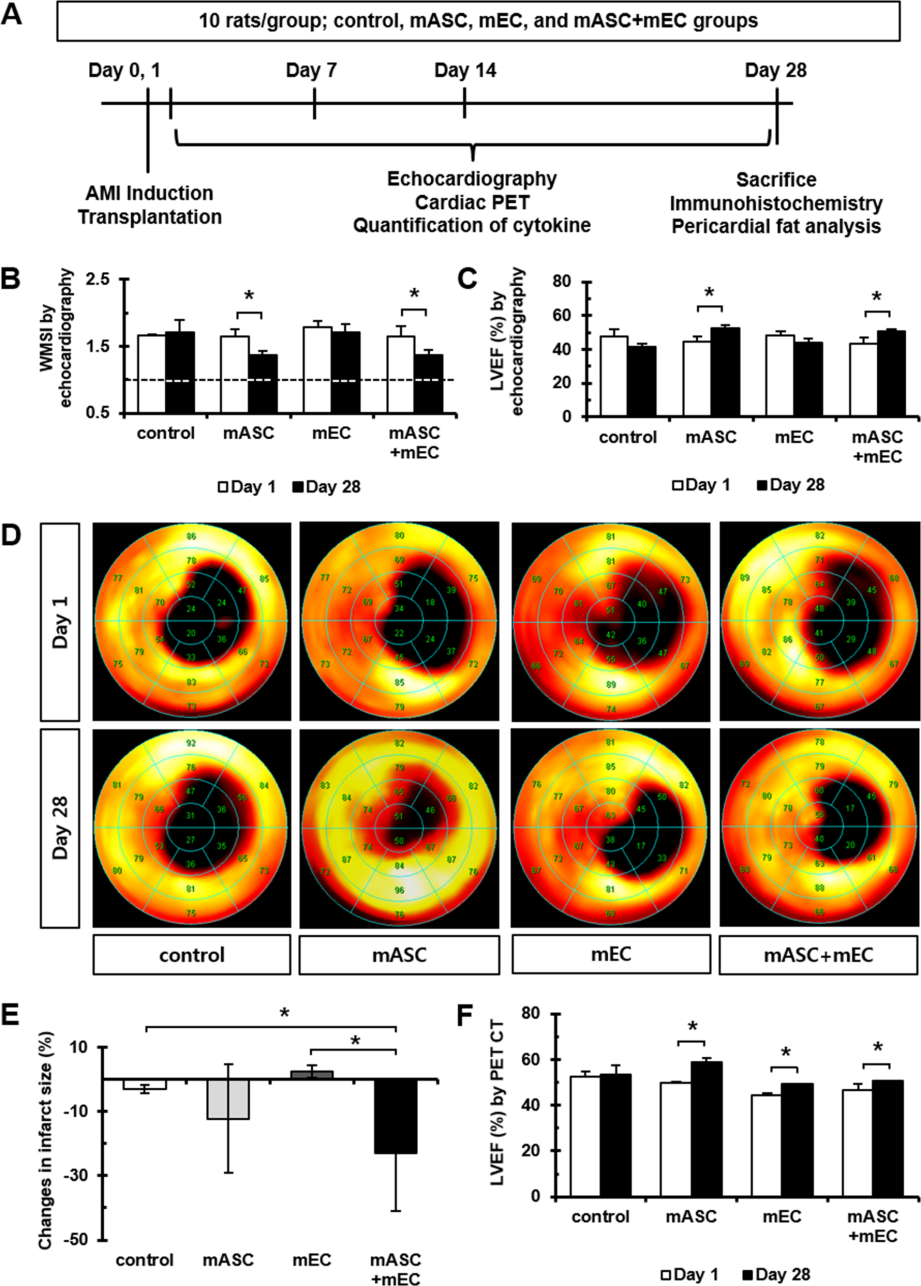
Recovery of myocardial function after cell transplantation. (A) Schematic diagram of study protocol. Ten rats were randomly assigned into the following 4 groups: control (only culture media), mASC (1 X 10^6^), mEC (1 X 10^6^), and mASC+mEC (5 X 10^5^ + 5 X 10^5^). (B) WMSI from echocardiography. A dotted line indicates the WMSI of non-infarcted hearts. *n* = 8, 10, 10, and 8 rats in each group, *p < 0.05. (C) EF was determined from 2D echocardiography. *n* = 8, 10, 10, and 8 rats in each group, *p < 0.05. (D) Representative image of a polarmap using the 20-segment Cedar-Sinai method with ^18^F-FDG PET. *n* = 4, 4, 4, and 5 rats in each group. (E) Changes in infarct size according to ^18^F-FDG PET polarmap. *n* = 4, 4, 4, and 5 rats in each group, *p < 0.05. (F) Changes in ejecting fraction according to ^18^F-FDG PET. *n* = 4, 4, 4, and 5 rats in each group, *p < 0.05.

Fifty-six rats were underwent AMI surgery. Among AMI induced rats, 10 rats for each group (control, mASC, mEC, and mASC+mEC) were assigned. In mortality and exclusion, 9 rats in control, mEC, and mASC+mEC groups (2, 4, and 3 rats, respectively) died during AMI surgery and post-operate care. Seven rats in control, mASC, mEC, and mASC+mEC groups (2, 1, 2, and 2 rats, respectively) were excluded due to an inordinately damage region (< 30% or > 60% of LVEF). Cardiac function was evaluated 28 days after transplantation of mASC and mEC into AMI induced rats (Fig 2A). Significant decreases in WMSI were observed at 28 days compared to 1 day in mASC and mASC+mEC groups (Fig 2B). In addition, mASC and mASC+mEC groups showed a significant increase in LVEF at 28 days (52.696 ± 1.849% and 50.520 ± 1.411%, respectively) compared to 1 day (44.505 ± 3.025% and 43.188 ± 3.743%, respectively). No statistically significant increase in LVEF was observed in control or mEC groups (Fig 2C) (S1A Fig).

Change of infarct size (%) was quantified based on images of PET scans taken at 1 and 28 days. Myocardial infarct size significantly decreased in mASC+mEC group compared to control and mEC groups. However, mASC group showed tendency of decreasing infarct size compared control group (Fig 2D and 2E). Increases in LVEF measured by PET were seen in mASC (9.1 ± 1.1%), mEC (5.0 ± 0.6%), and mASC+mEC groups (3.8 ± 2.4%) which were similar to echocardiography findings (Fig 2F). Cell transplantation therapies caused significant recovery of damaged myocardium compared to control group (1.1 ± 1.5%). Cross-sectional views from PET showed decreases in infarct size in mASC and mASC+mEC groups (S1B Fig).

### Comparison of fibrosis and α-SMA-positive vessel densities

**Fig 3:**
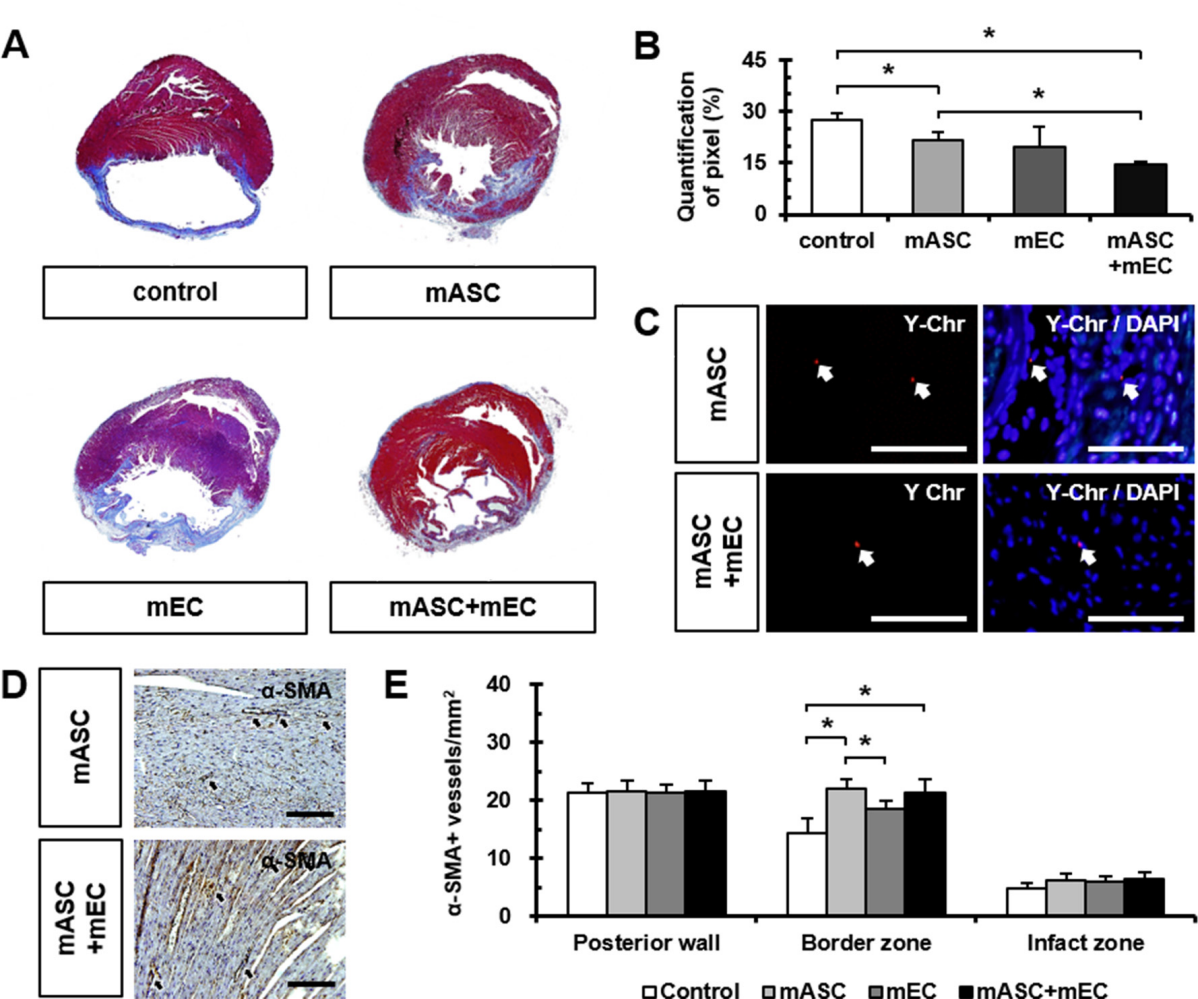
Comparison of fibrosis, small vessels densities, and engraftment after transplantation. (A) Representative MT stained images at papillary muscle levels showing myocardial fibrosis 28 days after AMI. *n* = 3 rats in each group. (B) Quantification of myocardial fibrosis after cell transplantation. *n* = 3 rats in each group, *p < 0.05. (C) Representative images of FISH staining for Y-Chr at sacrifice confirmed successful engraftment of male mASC in female AMI rats. *n* = 3 rats in each group. (D and E) Quantification of α-SMA-positive vessels in the posterior wall, border, and infarct zone. *n* = 5 rats in each group, *p < 0.05. Scale bar: 100 μm.

MT staining showed significant reduction in fibrosis after AMI in mASC (21.77 ± 4.62%) and mASC+mEC groups (14.74 ± 1.24%) compared to control group (27.43 ± 4.83%) (Fig 3A and 3B). Moreover, mASC which were obtained from male mice were detected in the recipient female rats by fluorescence staining for Y-Chr (Fig 3C).

α-SMA-positive vessels were counted at 3 areas: the posterior wall, border, and infarct zone. Significantly increases in the number of α-SMA-positive vessels were found in mASC (22.00 ± 1.70) and mASC+mEC groups (21.30 ± 2.41) compared to control (14.30 ± 2.71) in the border zone (Fig 3D and 3E). The numbers of α-SMA-positive vessels in mASC group were significantly increased compared to mEC group (18.60 ± 1.43) in the border zone (Fig 3E). No significant difference in the number of α-SMA-positive vessels was observed among the 4 groups in the posterior wall and infarct zone (Fig 3E). These results indicate that intramyocardially injected mASC and mASC+mEC promote local angiogenesis in the border zone.

### Increased pericardial fat after mASC transplantation

**Fig 4:**
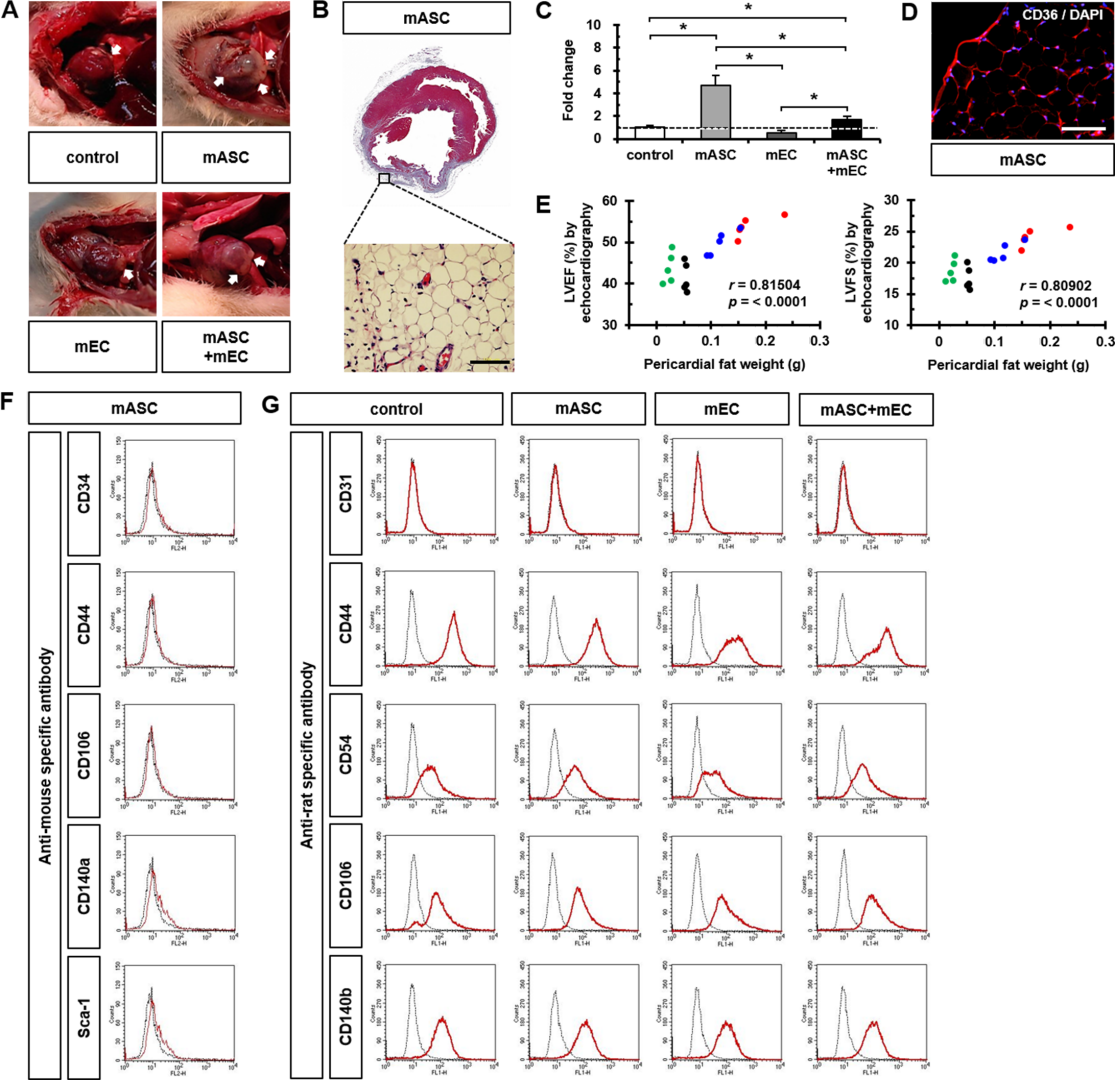
Identification of increased pericardial fat after cell transplantation. (A) Representative images of pericardial fat after cell transplantation. *n* = 10 rats in each group. (B) Identification of pericardial fat in mASC group (*n* = 3 rats) via magnified representative MT stained image. (C) Quantification of increased pericardial fat amount via IHC images. A dotted line indicates the value of five healthy rats (0.96 ± 0.18). *n* = 3 rats in each group, *p < 0.05. (D) Immunofluorescence images showing expression of the fat surface antigen FAT/CD36. Nuclei were stained with DAPI. *n* = 3 rats. (E) Positive correlations of pericardial fat weight with LVEF (left) or LVFS (right). Control = black, mASC = red, mEC = green, and mASC+mEC = blue dots. *n* = 5 rats in each group. (F and G) Flow cytometry analyses for characterization of mouse-specific and rat-specific surface antigens. *n* = 6 rats in each group, each measured in triplicate. Scale bar: 100 μm.

Pericardial fat observed at sacrifice was specifically located around the intramyocardial injection areas (Fig 4A), and MT staining confirmed adipose tissue in those areas (Fig 4B). Quantification of fat area based on H&E images demonstrated that mASC and mASC+mEC groups resulted in significant increases in pericardial fat compared to control (4.71-fold and 1.70-fold, respectively) or mEC groups (9.49-fold and 3.44-fold, respectively) (Fig 4C). Pericardial fat was positively stained for FAT/CD36, which is a known positive antigen for fat (Fig 4D). Pericardial fat weight was significantly higher in mASC and mASC+mEC groups (S2A Fig). However, there were no significant differences in body weight or heart weight of rats at sacrifice among four groups (S2B and S2C Fig). We found that pericardial fat weight showed significantly positive correlations with the parameters of cardiac function including LVEF and LVFS in control, mASC, mEC, and mASC+mEC groups (Fig 4E and S1 Table).

Flow cytometry with specific antibodies against fat surface antigens was used for identification of pericardial fat origin. Cells stained with mouse specific antibodies were not detected in pericardial fat (Fig 4F), but positive cells stained with rat specific antibodies such as CD44, CD54, CD106, and CD140b were found in pericardial fat (Fig 4G). FISH analysis did not identify a Y-Chr in pericardial fat tissue (data not shown). Thus, proliferated pericardial fat did not originate from the transplanted cells.

### Analysis of adipokines after cell transplantation

**Fig 5:**
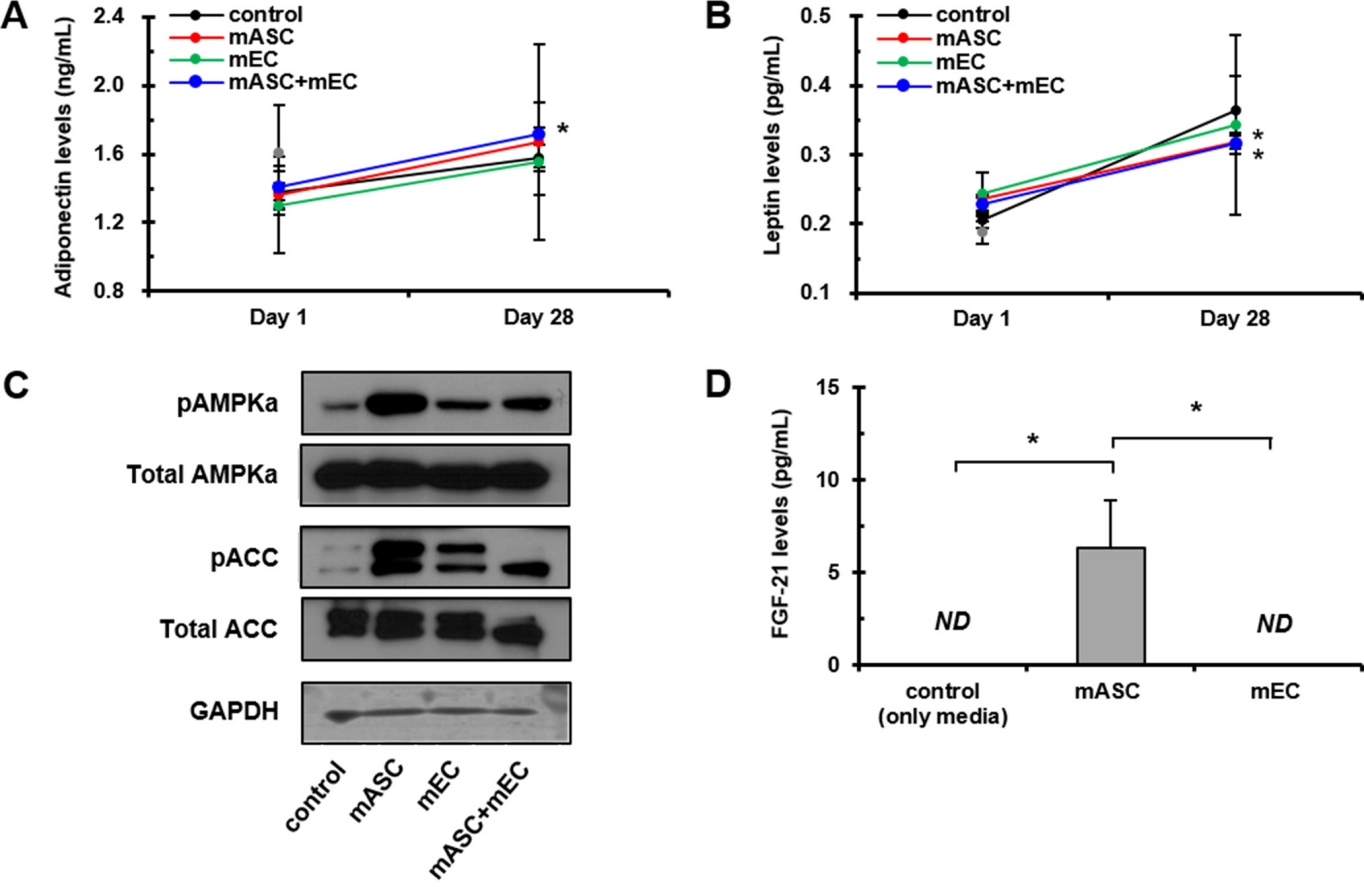
Secreted adipokines from pericardial fat after cell transplantation. (A and B) Changes in plasma adiponectin and leptin levels between day 28 and 1. Gray circles indicate plasma adiponectin (1.607 ± 0.274 ng/mL) and leptin levels (0.178 ± 0.015 pg/mL) in five healthy rats. *n* = 10 rats in each group, each measured in triplicate. *p < 0.05 *vs*. control. (C) Representative Western blot images of isolated pericardial fat-lysates for AMPK, pAMPKα, ACC, and pACC. *n* = 3 rats in each group, each measured in triplicate. (D) Secretion of FGF-21 from mASC and mEC. Evaluation of FGF-21 levels secreted from mASC and mEC after 24 hrs of seeding on 6-well plates. Only culture medium without cells was used as control. FGF-21 supernatant levels were significantly higher in mASC (6.364 ± 2.571 pg/mL) and were not detected (*ND*) in both control and mEC. Each sample was measured in triplicate. *p < 0.05.

A significant increase in plasma adiponectin levels between day 28 and 1 after AMI in mASC+mEC group (0.31 ± 0.021 ng/mL) was significantly higher than that of control group (0.20 ± 0.025 ng/mL) (Fig 5A). However, an increase in plasma adiponectin levels between day 28 and 1 after AMI in mASC group (0.31 ± 0.25 ng/mL) was not reached to the level of significance compared to that of control group (0.20 ± 0.02 ng/mL) may be due to a high variance (Fig 5A). Plasma adiponectin levels (1.607 ± 0.274 ng/mL) in the healthy rats were higher than plasma adiponectin levels (1.360 ± 0.214 pg/mL) at day 1 after AMI, but not significant. Significant increases in plasma leptin levels between day 28 and 1 after AMI in mASC (0.08 ± 0.003 pg/mL) and mASC+mEC group (0.09 ± 0.005 pg/mL) were found compared to that of control group (0.16 ± 0.025 pg/mL) (Fig 5B). Plasma leptin levels (0.178 ± 0.015 pg/mL) in the healthy rats were similar to plasma leptin levels (0.137 ± 0.016 pg/mL) at day 1 after AMI. Underlying mechanisms involved in the production of pericardial fat were further investigated. AMPK is an important molecule implicated in adipogenesis. pAMPK was significantly increased in mASC and mASC+mEC groups, and this increase in pAMPK levels are presumably influenced by various secreted adipokines. This active pAMPK is thought to inhibit ACC activity, thereby increasing pACC levels in mASC and mASC+mEC groups (Fig 5C). FGF-21, a master transcriptional regulator of adipogenesis, played a key role in insulin-sensitizing process in an adiponectin-dependent manner [15]. Therefore, we evaluated FGF-21 levels secreted from mASC and mEC by ELISA. Interestingly, we found that mASC abundantly secreted FGF-21 whereas mEC did not secrete FGF-21 (Fig 5D).

## Discussion

In a recent study, transplantation into cardiac disease models using ASC as well as mixtures of various cell sources has been attempted and yielded encouraging results [16]. ASC are well known for their superior capacity of vessel reparability and regeneration of myocardium after cell transplantation [17, 18]. We further investigated the synergistic reparative capacity of mASC+mEC in this study, and intramyocardial injections of both mASC and mASC+mEC into infarcted myocardium evidently resulted in increased LV contractile function, supported by PET and echocardiography analysis. PET was performed in this study to evaluate an objective quantitative assessment of LV function. However, the difference between echocardiography and PET results in mEC is due to excellent sensitivity and better spatial resolution of PET than echocardiography [19, 20]. These findings suggest that mASC are more dominantly involved in repair processes and exert superior regenerative capacity in damaged myocardium than mEC, which is terminally differentiated cells. Improvements in LV function after intramyocardial injection of mASC are supported by the reduced fibrosis with increases in small vessels densities observed in this study on 28 days.

It is generally accepted that pericardial fat is highly linked to impairment of decreased cardiac function in humans [21, 22]. Interestingly, pericardial fat increased prominently in mASC transplantation groups in the present study. Greater amounts of pericardial fat have generally been associated with increased risk for cardiovascular diseases [23, 24]. Even so, compensatory increases in pericardial fat after intramyocardial ASC injections could play a different role, such as facilitating improvement of LV function as seen in this study. By supporting the energy needs of arterial walls and myocardium and secreting fatty acids that prevent lipotoxicity [25], pericardial fat near ischemic tissue could reduce the degree of hypertrophy of the heart and thus facilitate a protective role in ischemia. The positive effects of increased adiponectin levels have been reported as enhancement of cardiac function in anesthetized pigs [26], and treatment with exogenous adiponectin which significantly reduces the infarct size in myocardial ischemia and reperfusion [27, 28]. Furthermore, high expression of adiponectin in patients with cardiac disease has been shown to provide protective local actions on adjacent coronary arteries [29].

In this study, transplantation of mASC not only increased the delta values of plasma adiponectin concentration, but also reduced the delta concentration in plasma leptin levels compared to control group. Our observation coincides with previous reports of correlations between plasma adipokine levels and heart function. A positive correlation between increases in plasma adiponectin levels and increases in hyperemic myocardial blood flow was found in morbidly obese individuals with surgical gastric bypass-induced weight loss [30]. Fontes-Carvalho *et al*. [31] also reported that patients with normal diastolic function had significantly lower leptin plasma levels compared to patients with diastolic dysfunction. In addition, increased leptin levels could result in hypertrophy of cardiomyocytes, increased incidence of AMI, aggravated atherosclerotic progression, or retarded recovery of damaged myocardium [32].

In terms of correlation between adipose tissue and cardiac function, Yuhki *et al*. [33] indicated that adiponectin secreted from adipose tissue activated AMPK signaling pathway, which increased energy supply resulting in preventing cell death in the ischemic heart, suggesting that adiponectin is a promising new therapy for AMI. Accordingly, it was necessary to study downstream events on cardiovascular system in response to changes of adiponectin and leptin levels secreted by pericardial fat. The changes in adiponectin and leptin levels were implicated by AMPK pathway and confirmed by Western blot results for AMPK and ACC. In this study, we observed that pAMPK levels in pericardial fat tissue were significantly increased as a result of mASC and mASC+mEC transplantation, indicating positive association with higher circulating adiponectin levels. In accordance with our observation, treatment of pioglitazone on obese rats has been shown to attenuate LV hypertrophy, fibrosis, and diastolic dysfunction by activating cardiac AMPK signaling as a result of the stimulation of adiponectin secretion [34]. Pei *et al*. [35] also reported that cardiac-derived adiponectin induced by long-term insulin treatment ameliorated myocardial ischemia/reperfusion injury in type 1 diabetic mice via myocardial AMPK activation. In addition, adiponectin improved cardiac function through anti-apoptotic effects caused by up-regulation of AMPK in doxorubicin-induced cardiomyopathy in mice [36]. A number of studies have reported that AMPK phosphorylates both ACC1/2 on serine residues, resulting in inactivation of ACC for control of lipid metabolism [37, 38]. Similarly, we also found that phosphorylation of ACC in pericardial fat tissues significantly increased in mASC and mASC+mEC groups, demonstrating that ACC is a downstream target of AMPK [39, 40]. It seems that implantation of ASC results in increased pericardial fat that secretes higher levels of adiponectin. Higher adiponectin secretion activates pAMPK in the infarcted heart, and in turn activated ACC is inactivated via phosphorylation induced by pAMPK [41]. Regulation of fatty acid synthesis and oxidation via AMPK results in the enhancement in heart function [42].

To confirm that the engraftment was successfully introduced, the infarcted myocardium was subjected to fluorescence staining for Y-Chr. Although direct intramyocardial injection is known for its low retention rate and high death rates of transplanted cells, we detected several transplanted mASC by extensive FISH analysis within the peri-infarct areas on 28 days. Cell fusion between donor cells and transplanted cells was not observed in this study. Anti-rat specific antibody binding was detected in pericardial fat, while anti-mouse specific antibody binding was not, indicating that pericardial fat generated after cell transplantation originated from the host rat and was not proliferated tissue from the donor cells. These results suggest that injected mASC exert positive paracrine effects on the infarcted pericardium with increased production of a protective layer, ultimately resulting in improved myocardial function.

In this study, we found that mASC abundantly secreted FGF-21 whereas mEC did not secrete FGF-21 (Fig 5D). It is quite possible that mASC-secreted FGF-21 could contribute to formation of pericardial fat after mASC injection, finally resulting in the improvement of cardiac function. In accordance with these results, increased pericardial fat weight via transplantation of mASC showed a significant positive association with the improvement in cardiac function such as LVEF and LVFS measured by echocardiography at 28 days (Fig 4E). The correlation of pericardial fat with body weight, heart weight, or deposition of visceral fat was examined, but these did not significantly affect each other in this study.

In our study, although xenograft transplantation was performed by implantation of mASC into AMI rat models, we considered immunosuppression overcome by following reasons. Xenograft transplantation of MSC including ASC facilitates homing of cells to the ischemic site and induces immune tolerance to suppress rejection response to xenograft transplantation [43, 44]. However, recipient immune responses after ASC and EC transplantation into AMI models of immunodeficient animals should be further investigated. In the future, additional investigation about changes in various adipokines such as resistin, visfartin, chemerin, and apelin in plasma should be helpful.

## Conclusions

In conclusion, this study demonstrated enhanced LV function upon intramyocardial injection of mASC after AMI in rats with increased pericardial fat. Increased circulating adiponectin levels together with decreased leptin levels after intramyocardial ASC injections suggest beneficial paracrine effects of secondary increase in pericardial fat in AMI models. Increased adiponectin secretion from pericardial fat with its association with enhanced LV function could provide new clinical implications of ASC transplantation in AMI.

## Supporting Information

S1 Fig Cardiac functional recovery by cell transplantation. (A) Representative images of two-dimensional echocardiography obtained at 28 days after cell transplantation. Anterior systolic wall thickness was significantly higher in cell transplantation groups than control group. *n* = 8, 10, 10, and 8 rats in each group. (B) Representative AMI model ^18^F-FDG PET images showing short axis (SA), horizontal long axis (HLA), and vertical long axis (VLA) views of all groups. Distinctive recovery of myocardium was observed in mASC and mASC+mEC groups. *n* = 4, 4, 4, and 5 rats in each group.
(TIF)

S2 Fig Parameter analysis after cell transplantation. (A) Comparisons between pericardial fat weights at 28 days after cell transplantation of all groups. Significantly increased pericardial fat weight was observed in mASC and mASC+mEC groups compared with control and mEC groups. A dotted line indicates the value of healthy rats (0.06 ± 0.01 g). *n* = 5 rats in each group, *p < 0.05. (B) No significant differences in heart weight were observed between any groups at 28 days after cell transplantation. A dotted line indicates the value of healthy rats (0.86 ± 0.04 g). *n* = 5 rats in each group, *P*-value not significant (*NS*). (C) Comparison of body weight between 0 and 28 days. No significant differences in body weight change were observed in any groups. A dotted line indicates the value of five healthy rats (208.9 ± 3.20 g and 247.5 ± 8.90 g). *n* = 10 rats in each group, *NS*.
(TIF)

S1 Table Correlations of pericardial fat weight with cardiac function. (TIF)
