# Supplementary figures and images for: Intramyocardial Adipose-Derived Stem Cell Transplantation Increases Pericardial Fat with Recovery of Myocardial Function after Acute Myocardial Infarction
Source: PLoS One. 2016 Jun 23;11(6):e0158067. doi: 10.1371/journal.pone.0158067 (PMC4919032; doi:10.1371/journal.pone.0158067)

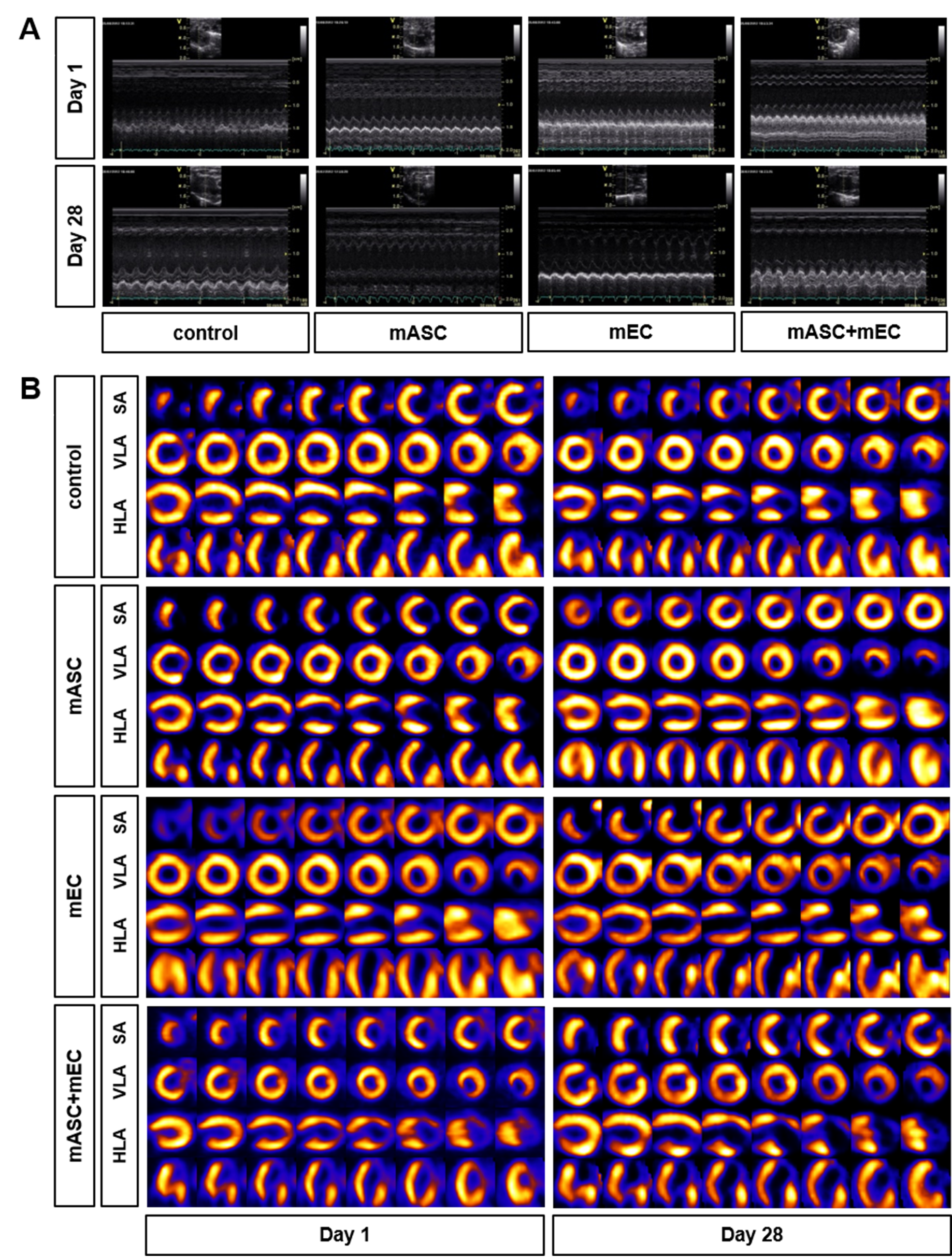

Supplement: S1 Fig — (A) Representative images of two-dimensional echocardiography obtained at 28 days after cell transplantation. Anterior systolic wall thickness was significantly higher in cell transplantation groups than control group. n = 8, 10, 10, and 8 rats in each group. (B) Representative AMI model 18F-FDG PET images showing short axis (SA), horizontal long axis (HLA), and vertical long axis (VLA) views of all groups. Distinctive recovery of myocardium was observed in mASC and mASC+mEC groups. n = 4, 4, 4, and 5 rats in each group. (TIF) [file pone.0158067.s001.tif]

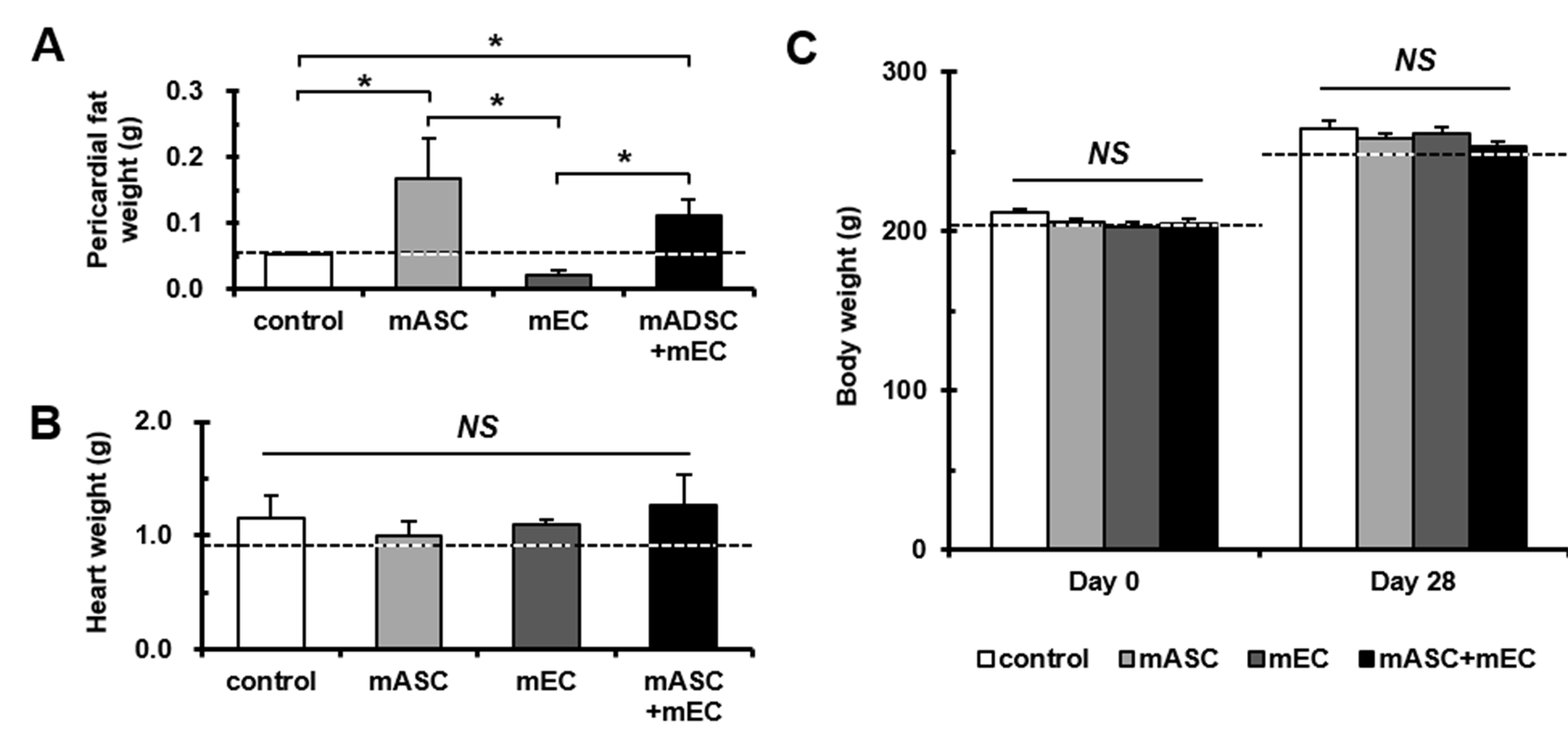

Supplement: S2 Fig — (A) Comparisons between pericardial fat weights at 28 days after cell transplantation of all groups. Significantly increased pericardial fat weight was observed in mASC and mASC+mEC groups compared with control and mEC groups. A dotted line indicates the value of healthy rats (0.06 ± 0.01 g). n = 5 rats in each group, *p < 0.05. (B) No significant differences in heart weight were observed between any groups at 28 days after cell transplantation. A dotted line indicates the value of healthy rats (0.86 ± 0.04 g). n = 5 rats in each group, P-value not significant (NS). (C) Comparison of body weight between 0 and 28 days. No significant differences in body weight change were observed in any groups. A dotted line indicates the value of five healthy rats (208.9 ± 3.20 g and 247.5 ± 8.90 g). n = 10 rats in each group, NS. (TIF) [file pone.0158067.s002.tif]

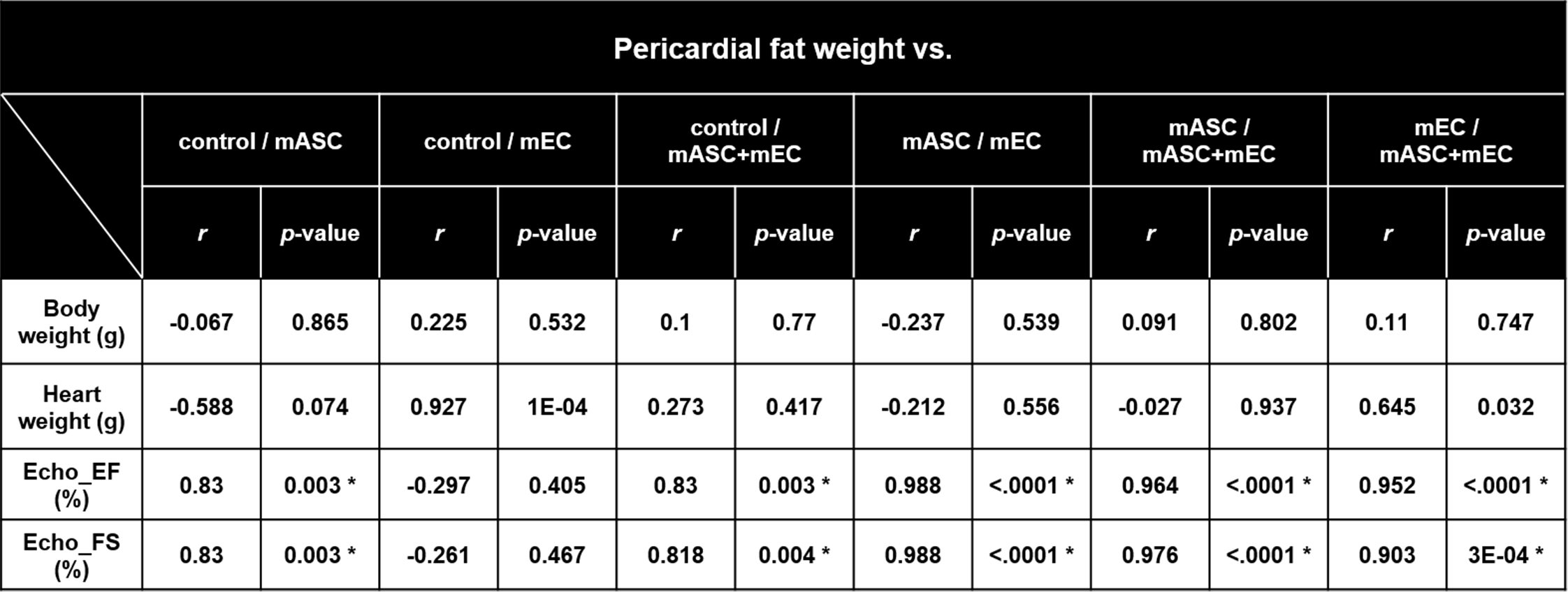

Supplement: S1 Table — (TIF) [file pone.0158067.s003.tif]
